# Role of Tumor Necrosis Factor-α and Natural Killer Cells in Uterine Artery Function and Pregnancy Outcome in the Stroke-Prone Spontaneously Hypertensive Rat

**DOI:** 10.1161/HYPERTENSIONAHA.116.07933

**Published:** 2016-10-12

**Authors:** Heather Yvonne Small, Ryszard Nosalski, Hannah Morgan, Elisabeth Beattie, Tomasz J. Guzik, Delyth Graham, Christian Delles

**Affiliations:** From the BHF Glasgow Cardiovascular Research Centre, Institute of Cardiovascular and Medical Sciences, University of Glasgow, Scotland (H.Y.S., R.N., H.M., E.B., T.J.G., D.G., C.D.); and Department of Internal Medicine, Jagiellonian University Medical College, Kraców, Poland (R.N.).

**Keywords:** blood pressure determination, etanercept, hypertension, pregnancy outcome, uterine artery

## Abstract

Women with chronic hypertension are at increased risk of maternal and fetal morbidity and mortality. We have previously characterized the stroke-prone spontaneously hypertensive rat (SHRSP) as a model of deficient uterine artery function and adverse pregnancy outcome compared with the control Wistar–Kyoto. The activation of the immune system plays a role in hypertension and adverse pregnancy outcome. Therefore, we investigated the role of tumor necrosis factor-α in the SHRSP phenotype in an intervention study using etanercept (0.8 mg/kg SC) at gestational days 0, 6, 12, and 18 in pregnant SHRSP compared with vehicle-treated controls (n=6). Etanercept treatment significantly lowered systolic blood pressure after gestational day 12 and increased litter size in SHRSP. At gestational day 18, etanercept improved the function of uterine arteries from pregnant SHRSP normalizing the contractile response and increasing endothelium-dependent relaxation, resulting in increased pregnancy-dependent diastolic blood flow in the uterine arteries. We identified that the source of excess tumor necrosis factor-α in the SHRSP was a pregnancy-dependent increase in peripheral and placental CD3^–^ CD161^+^ natural killer cells. Etanercept treatment also had effects on placental CD161^+^ cells by reducing the expression of CD161 receptor, which was associated with a decrease in cytotoxic granzyme B expression. Etanercept treatment improves maternal blood pressure, pregnancy outcome, and uterine artery function in SHRSP by antagonizing signaling from excess tumor necrosis factor-α production and the reduction of granzyme B expression in CD161^+^ natural killer cells in SHRSP.

## Abstract 2

Supplemental Digital Content is available in the text.

**See Editorial Commentary, pp 1108–1109**

Chronic hypertension in pregnancy is defined by the American College of Obstetricians and Gynaecologists as systolic blood pressure (SBP) >140 mm Hg and/or diastolic blood pressure >90 mm Hg, which has presented before pregnancy or before gestational week 20.^1^ Despite affecting a similar number of women as other hypertensive disorders of pregnancy, maternal chronic hypertension receives relatively less research attention.^2^ Women with chronic hypertension are at increased risk of maternal and fetal morbidity and mortality.^3^ The problem of chronic hypertension in pregnancy is growing in parallel to the prevalence of increased maternal age,^4^ obesity,^5^ and metabolic syndrome.^6^ There is no consensus on the treatment of pregnant women with chronic hypertension and no generally accepted target blood pressure at which the risk of adverse complications to both mother and fetus is substantially reduced.^7^ Research into the cardiovascular complications associated with pregnancy in women with pre-existing hypertension is therefore needed.

Vascular remodeling plays a central role in maternal adaptation to pregnancy to accommodate the increase in cardiac output and regulate maternal blood pressure.^8^ Specifically, the uterine vasculature must undergo extensive structural and functional changes to provide sufficient blood supply to the developing placenta and fetus. It is well established that deficient adaptation of the uterine vasculature leads to adverse pregnancy outcomes.^9^ We recently hypothesized that pre-existing maternal cardiovascular disease would negatively affect the ability of the vasculature to suitably adapt to pregnancy and tested this hypothesis in the stroke-prone spontaneously hypertensive rat (SHRSP). The SHRSP is a well-established rat model that mirrors many features of human hypertension-related cardiovascular complications and has been used in research for >30 years.^10^ Some aspects of pregnancy in the SHRSP have already been briefly characterized^11,12^; however, the vascular adaptation to pregnancy has not been studied systematically. We have shown that the SHRSP presents with reduced litter size and increased loss of glycogen storage from the placenta associated with spontaneous deficient uterine artery remodeling and blood flow relative to the contrasting strain, Wistar–Kyoto (WKY), at gestational day (GD) 18.^13^

Activation of the innate immune system has been reported in both chronic hypertension^14^ and adverse pregnancy outcome.^15^ Levels of the proinflammatory cytokine tumor necrosis factor-α (TNF-α) have been found to be increased in hypertensive subjects^16^ and in women with severe hypertensive pregnancy complications.^17^ Furthermore, TNF-α infusion induces vascular dysfunction in both humans and rodents.^18,19^ During pregnancy, TNF-α can be produced from a plethora of cell types but predominantly by activated monocytes/macrophages, T lymphocytes, natural killer (NK) cells, and invasive fetally derived extravillous trophoblasts.^20^ Antagonism of TNF-α signaling has not been used clinically to treat hypertension in pregnancy but has been shown to have therapeutic effects in preclinical models of pregnancy-induced hypertension.^21,22^

Here, we hypothesize that TNF-α provides a link between vascular dysfunction and adverse pregnancy outcome by playing a causative role in the abnormal uterine vascular function and reduced litter size in the SHRSP at GD 18. We performed an intervention study using etanercept in pregnant SHRSP and sought to identify the source of excess TNF-α.

## Methods

Most of the methods have been previously described by our group including the implantation of radiotelemetry probes,^23^ Doppler ultrasound,^13^ myography of the uterine arteries,^13,24^ and periodic acid–Schiff stain of the placenta.^13^ Full details are found in the online-only Data Supplement.

### Animals

Animals (SHRSP and WKY) were housed under controlled lighting (0700–1900 hours) and temperature (21±3 °C) and received a normal diet (rat and mouse no.1 maintenance diet; Special Diet Services, Grangemouth, United Kingdom) provided ad libitum. All animal procedures were approved by the Home Office according to the regulations on experiments with animals in the United Kingdom (Project License Number 60/4286). Females were time-mated at 12 weeks of age (±4 days). Non-pregnant animals were age-matched at 15 weeks±4 days (ie, 12 weeks of age+21 days of pregnancy). Day 0 of pregnancy was defined as the day that a coital plug was observed indicative of successful mating having taken place. The number of rats used for a given experiment is indicated in the associated figure legend.

### Etanercept Treatment

Animals were treated with etanercept 0.8 mg/kg (Wyeth Pharmaceuticals, Maidenhead, United Kingdom) diluted in sterile phosphate-buffered saline (Thermo Fisher Scientific, Paisley, United Kingdom) on GD 0, 6, 12, and 18 of pregnancy via SC injection. Vehicle-treated animals were subject to the same procedure using sterile phosphate-buffered saline (Thermo Fisher Scientific, Paisley, United Kingdom).

### Data Analysis

Analysis of flow cytometry data was performed using FlowJo v.10 where gates were applied according to fluorescence minus one control. For quantitative real-time PCR data, results were analyzed using the 2^−ΔΔCT^ method. Statistical analysis was performed using GraphPad Prism v 4.0. Student *t* test and 1-way ANOVA followed by Tukey test were used to compare WKY and SHRSP and WKY, SHRSP, and SHRSP treated with etanercept. Radiotelemetry data were analyzed using 2-way ANOVA, and the difference in blood pressure between SHRSP and SHRSP treated with etanercept was analyzed by calculating the ΔSBP from GD 12–GD 21 followed by Student *t* test. *P* <0.05 was considered to be statistically significant for all experiments.

## Results

### TNF-α Is Increased in Pregnant SHRSP Relative to WKY

**Figure 1.**
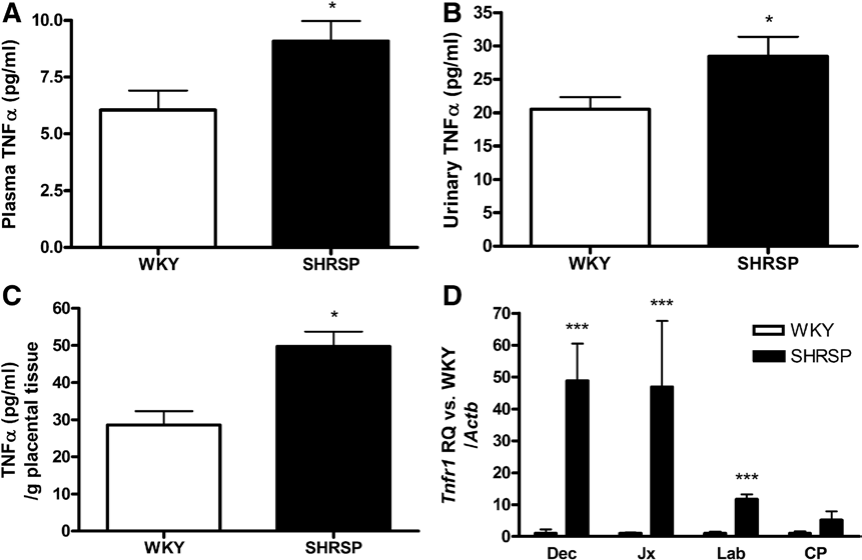
Tumor necrosis factor-α (TNF-α) is increased in pregnant stroke-prone spontaneously hypertensive rat (SHRSP). TNF-α was measured in plasma (**A**) and urine (**B**) using ELISA (n=6; *P<0.05 vs Wistar–Kyoto [WKY]). Secretion of TNF-α (**C**) was increased from placental explants (n=5; *P<0.05 vs GD 18 WKY). Gene expression of TNF-α receptor-1 (*Tnfr1*; **D**) was measured in the uteroplacental unit (n=6; ***P<0.005 vs GD 18 WKY). Data were analyzed by Student *t* test. CP indicates chorionic plate; Dx, decidua; Jx, junctional zone; and Lx, labyrinth.

TNF-α was increased in plasma and urine from pregnant (GD 18) SHRSP relative to pregnant WKY (Figure 1A and 1B). Increased secretion of TNF-α was also detected in media taken from placental tissue explants from GD 18 SHRSP (Figure 1C). Etanercept treatment in SHRSP did not significantly alter TNF-α levels from GD 18 plasma samples or placental explant media (Figure S1). Gene expression of the main proinflammatory TNF-α receptor-1 (*Tnfr1*) was also significantly increased in the GD 18 uteroplacental unit from SHRSP relative to WKY (Figure 1D).

### Etanercept Treatment Reduces Systolic Blood Pressure in the Pregnant SHRSP

**Figure 2.**
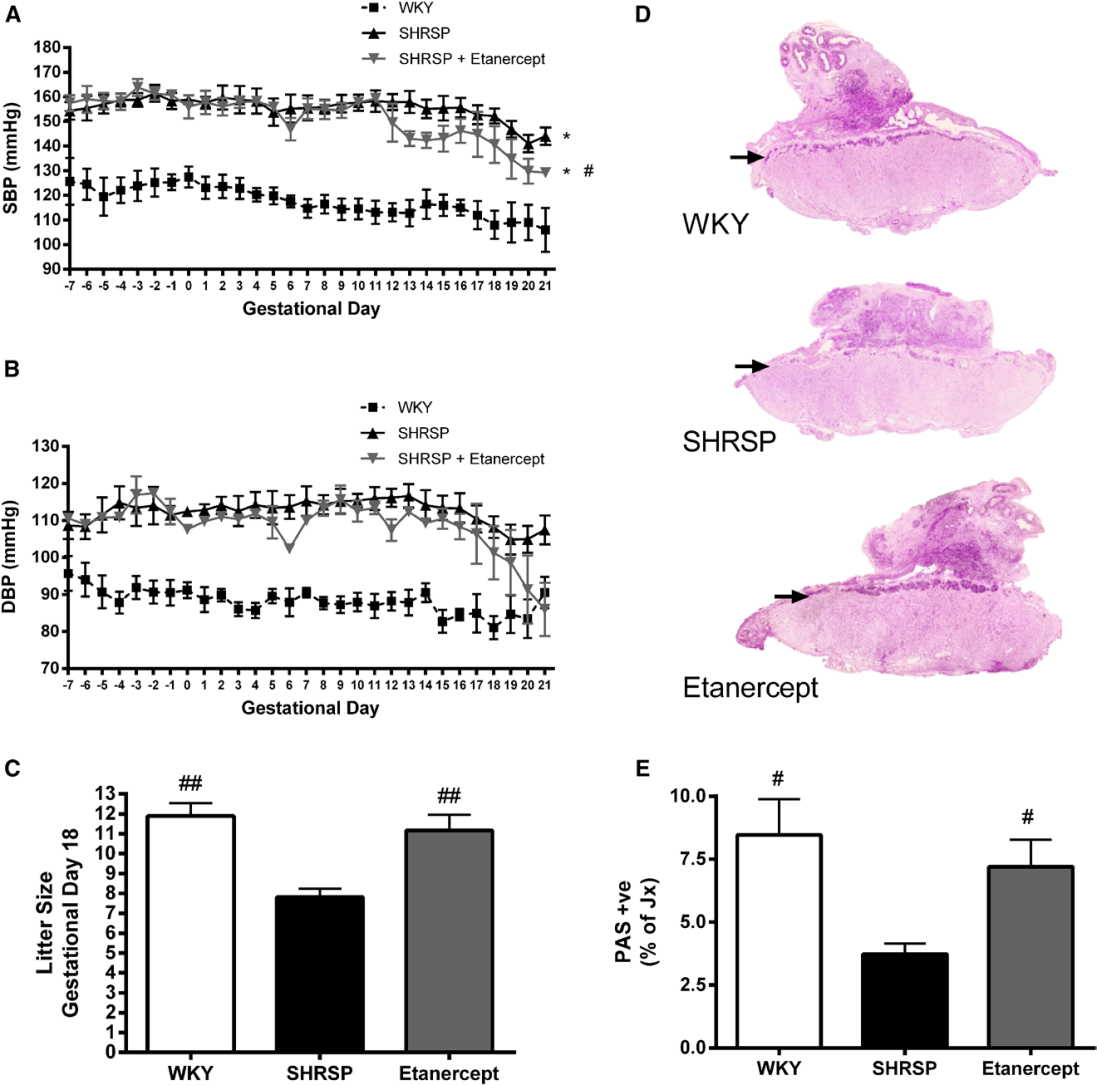
Etanercept treatment reduces systolic blood (SBP) pressure and improves placental biology and pregnancy outcome in the stroke-prone spontaneously hypertensive rat (SHRSP). SBP (**A**) and diastolic blood pressure (DBP; **B**) were monitored using radiotelemetry in Wistar–Kyoto (WKY), SHRSP, and etanercept-treated SHRSP (n=4–6) from 7 d before pregnancy (gestational day [GD]−7 to −1) and during pregnancy (GD 0–21). SHRSP and SHRSP treated with etanercept had significantly increased SBP and DBP relative to the WKY from GD−7 to GD 21 (*P<0.05 vs WKY analyzed by 2-way ANOVA). Etanercept-treated SHRSP had a significant decrease in SBP from GD 12–21 relative to vehicle-treated SHRSP (#*P*<0.05 vs SHRSP analyzed by ΔSBP GD 12–21 followed by a Student *t* test). Glycogen cell content in the junctional zone (Jx) of the placenta was assessed by counting the number of periodic acid–Schiff –positive cells in WKY, SHRSP, and SHRSP treated with etanercept (n=6). A representative uteroplacental unit for each group is shown in **C** and quantified in **E** (#*P*<0.05 vs SHRSP analyzed by 1-way ANOVA followed by a post hoc Tukey test). **D**, Litter size was counted at GD 18 in WKY, SHRSP, and etanercept (n=6–12; ##*P*<0.01 vs SHRSP analyzed by 1-way ANOVA followed by a post hoc Tukey test).

Etanercept (0.8 mg/kg SC) was given at GD 0 of pregnancy and repeated at GD 6, 12, and 18, and the effect on blood pressure in the SHRSP relative to vehicle-treated controls was monitored using radiotelemetry. SHRSPs are hypertensive before and during pregnancy in contrast to the control WKY strain (Figure 2A and 2B). Etanercept had no effect on SHRSP blood pressure in early pregnancy (GD 0–12). After GD 12, there was a significant average decrease of 11.5 mm Hg in SBP in etanercept relative to vehicle-treated SHRSP (Figure 2A), which persisted until parturition. No significant difference between SHRSP and etanercept-treated SHRSP was observed in diastolic blood pressure (Figure 2B). Heart rate was not significantly different between groups (Figure S2A). Activity was significantly decreased in both SHRSP and SHRSP treated with etanercept relative to the WKY over the course of pregnancy (Figure S2B).

### Etanercept Improves Placental Abnormality and Litter Size in the SHRSP

Placenta from SHRSP exhibited a significant decrease in glycogen storage at GD 18 relative to the WKY (Figure 2C through 2E). Etanercept treatment in the SHRSP restored periodic acid–Schiff–positive glycogen cells within the junctional zone of the placenta (Figure 2C through 2E). Litter size was significantly increased by etanercept treatment in SHRSP (Figure 2D). Concurrently, the number of dams, which presented with ≥1 resorptions, was decreased in SHRSP treated with etanercept (WKY, 30%; SHRSP, 66.7%; SHRSP treated with etanercept, 25%). Litter size was not related to the changes in blastocyst implantation as there was no difference in the number of implantation sites at GD 6 between the groups (Figure S3). Furthermore, there was no difference in fetal or placental weight between any of the groups (Figure S4).

### Etanercept Improves Abnormal Uterine Artery Function and Blood Flow in the Pregnant SHRSP

**Figure 3.**
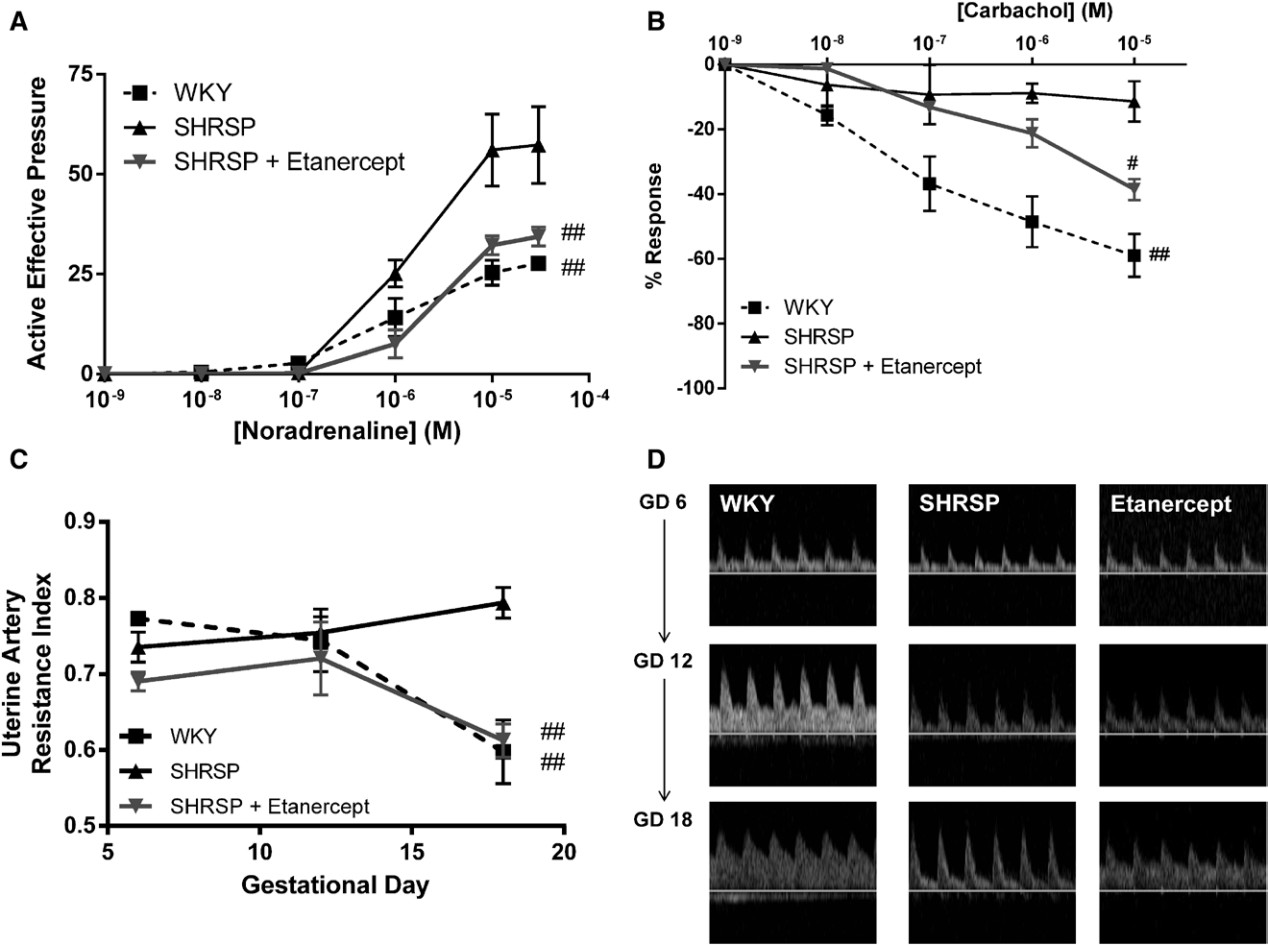
Etanercept treatment significantly improves gestational day (GD) 18 uterine artery function and blood flow in the stroke-prone spontaneously hypertensive rat (SHRSP). Isolated uterine artery function was assessed using wire myography in Wistar–Kyoto (WKY), SHRSP, and SHRSP treated with etanercept (n=6) at GD 18 of pregnancy. Uterine artery contractile response was determined using noradrenaline (NA; **A**), and endothelium-dependent vasorelaxation was assessed using carbachol response (**B**). Uterine arteries from etanercept-treated SHRSP had a significantly reduced contractile response to NA (**A**) relative to SHRSP (##*P*<0.01 vs SHRSP analyzed by calculating the area under the curve followed by 1-way ANOVA) and a significantly increased relaxation response to carbachol (**B**) at 1×10^−^^5 mol/L^ (#*P*<0.05 vs SHRSP analyzed by Student *t* test). Uterine artery blood flow was assessed using Doppler ultrasound in WKY, SHRSP, and SHRSP treated with etanercept (n=6) at GD 6, 12, and 18. Representative Doppler traces are shown in (**C**). The pregnancy-induced increase in diastolic blood flow which is present in the WKY but reduced in SHRSP is partially restored in SHRSP treated with etanercept (**C**) resulting in a significant reduction in resistance index at GD 18 (**D**; ##*P*<0.01 vs SHRSP analyzed by 1-way ANOVA followed by a post hoc Tukey test).

Pressure myography was used to construct pressure–diameter relationships to assess uterine artery properties from GD 18 WKY, SHRSP, and SHRSP treated with etanercept. Etanercept treatment did not significantly alter the diameter or wall thickness of the SHRSP uterine arteries (Figure S5). Investigation of uterine artery vasomotor function using wire myography showed that uterine arteries from pregnant SHRSP had a significantly increased contractile response to noradrenaline and a blunted endothelium-dependent vasorelaxation relative to the WKY (Figure 3A and 3B). In contrast, uterine arteries from pregnant SHRSP treated with etanercept exhibited a marked reduction in contractile response that was not significantly different from WKY and a significant increase in vasorelaxation to carbachol (Figure 3A and 3B). Etanercept treatment did not have a significant effect on mesenteric artery function in SHRSP (Figure S6).

These changes in uterine artery vasomotor function translated into improvements in uterine artery blood flow were assessed by Doppler ultrasound. Etanercept treatment partially restored the physiological increase in diastolic volume over the course of pregnancy, which is present in the WKY but absent in the SHRSP (Figure 3C), resulting in significantly reduced uterine artery resistance index in etanercept-treated SHRSP relative to control SHRSP (Figure 3D).

### NK Cells Are a Source of Excess TNF-α in the SHRSP

**Figure 4.**
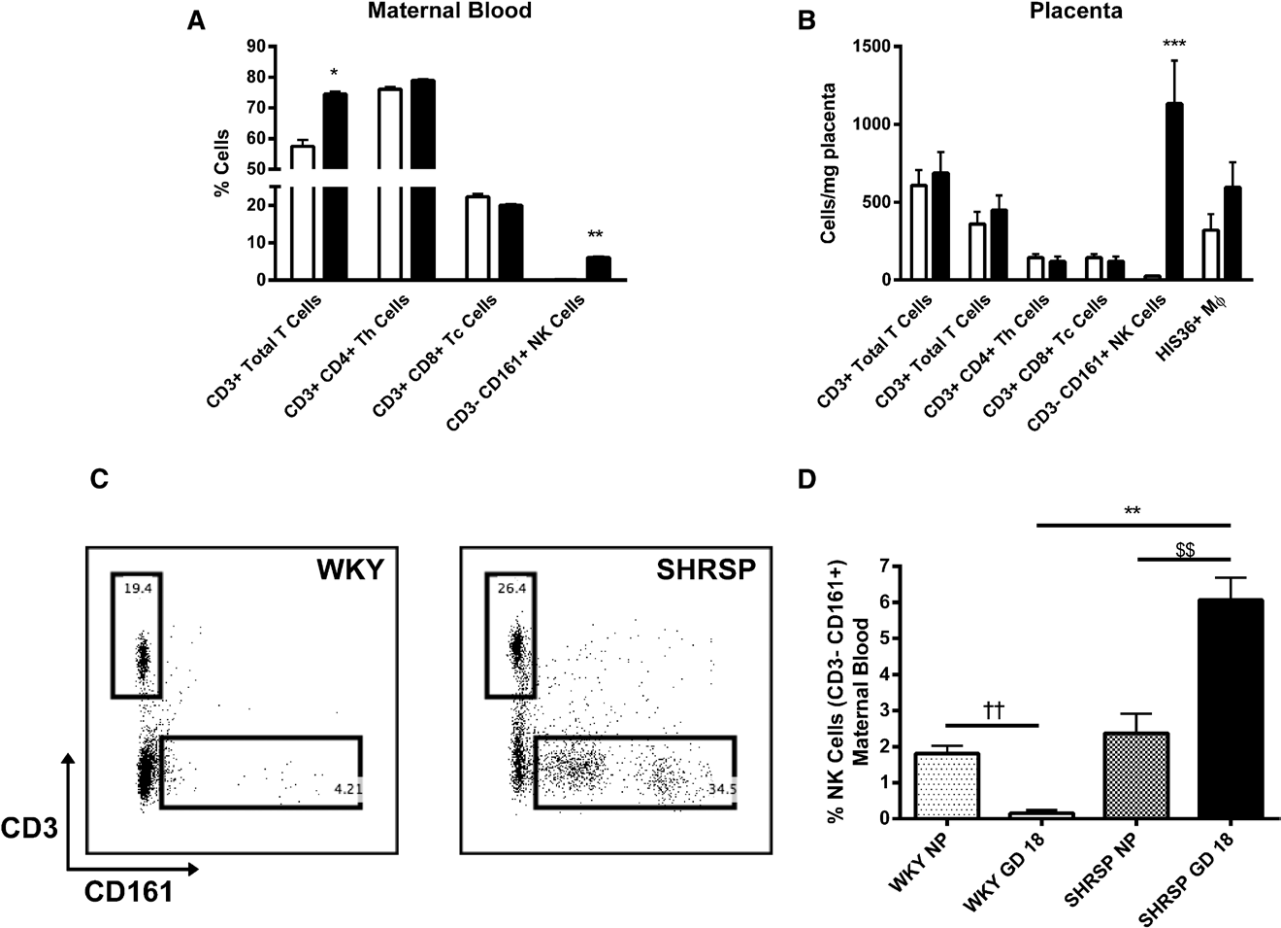
CD161^+^ natural killer (NK) cells are significantly increased in pregnant stroke-prone spontaneously hypertensive rat (SHRSP). Flow cytometry was used to measure leukocyte populations in maternal blood (**A**) and placenta (**B**) from GD 18 Wistar–Kyoto (WKY) and SHRSP (n=5–6). CD3^+^ T cells and CD3^−^ CD161^+^ NK cells were elevated in maternal blood from SHRSP relative to WKY (**A**; *P<0.05 vs WKY; **P<0.01 vs WKY). CD3^−^ CD161^+^ NK cells were significantly increased in placenta from SHRSP relative to WKY (**B**). A representative dot plot showing the increase in CD3^−^ CD161^+^ cells in placenta from SHRSP relative to WKY is shown in (**C**). NK cell numbers were assessed using flow cytometry in virgin (NP; n=4) and GD 18 (n=5–6) SHRSP and WKY (**D**; ††*P*<0.01 vs WKY nonpregnant [NP]; $$*P*<0.01 vs SHRSP NP). Data were analyzed by 1-way ANOVA followed by post hoc Tukey test. Th indicates T helper cell; and Tc, cytotoxic T cell.

From the beneficial effects we observed in pregnancy outcome and uterine artery function in SHRSP treated with etanercept, we deduced that excess TNF-α signaling played a causal role in the pathology observed during pregnancy in this model. Although TNF-α can be produced in some quantity from almost all cell types, we focused on one of the major producers, the immune cells, to identify the source(s) of excess TNF-α in the SHRSP. We designed a flow cytometry panel to quantify immune cell populations in pregnant WKY and SHRSP in the maternal blood and placenta (Figure S7 and S8, full panel and Figure S9, gating strategy). Of the populations analyzed, NK cells (CD3^−^ CD161^+^) were the most markedly increased in the SHRSP relative to WKY in both maternal circulation (Figure 4A) and placenta (Figure 4B and 4C). Furthermore, we detected pregnancy-specific changes in peripheral CD3^−^ CD161^+^ cells in maternal blood. In WKY, these cells were significantly decreased from nonpregnant to pregnant (GD 18) WKY (Figure 4D). In contrast, there was a significant increase in peripheral NK cells in SHRSP from nonpregnant to GD 18 (Figure 4D).

**Figure 5.**
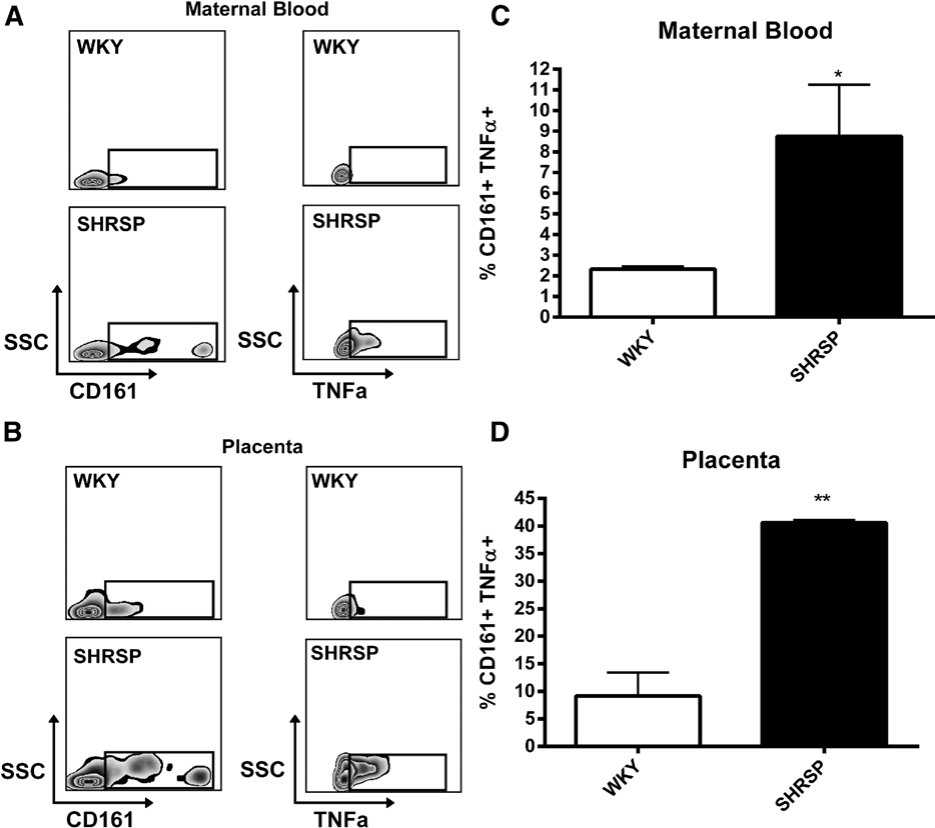
CD161^+^ cells are a source of excess TNF-α in pregnant SHRSP maternal circulation and placenta. Intracellular flow cytometry was used to measure TNF-α production in CD161^+^ natural killer cells in maternal blood (**A**–**B**) and placenta (**C**–**D**) from GD 18 WKY and SHRSP (n=4). CD161^+^ cells from the maternal circulation (**A**–**B**) and placenta (**C**–**D**) showed an increased production of TNF-α from pregnant SHRSP relative to WKY (* *P*<0.05; **P<0.01 vs WKY GD 18). Data were analyzed by Students *t* test.

The significant increase in NK cells in the maternal circulation and placenta of the SHRSP was a promising candidate as the source of excess TNF-α. Intracellular staining of CD161^+^ cells in both the maternal circulation (Figure 5A and 5B) and placenta (Figure 5C and 5D) showed that TNF-α production is significantly increased in the SHRSP relative to the WKY at GD 18. TNF-α production from CD3^+^ T cells or other CD45^+^ cells was not significantly different between the strains (Figure S10).

### Etanercept Reduces Granzyme B Production From CD161^+^ Cells in the SHRSP Placenta

**Figure 6.**
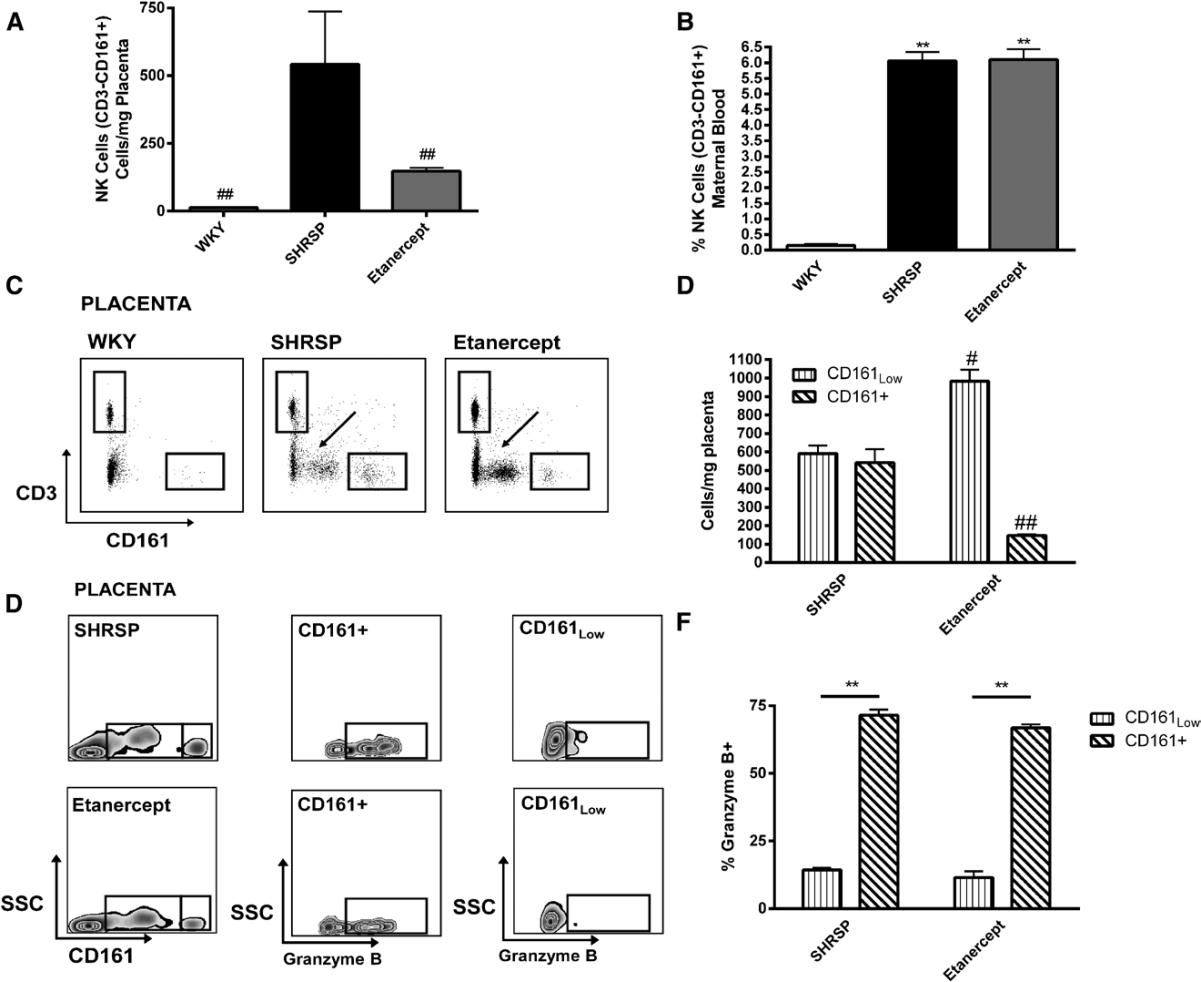
Etanercept reduces granzyme B–producing CD161^+^ cells in the SHRSP placenta. Flow cytometry was used to quantify CD3^−^ CD161^+^ NK cells in SHRSP and SHRSP treated with etanercept (n=5–6). Etanercept treatment significantly decreased CD3^−^ CD161^+^ cells in the SHRSP GD 18 placenta (**A**; ##*P*<0.01 vs SHRSP GD 18) but not in maternal blood (**B**). **C**–**D**, Etanercept treatment caused a shift in CD161^+^ expression from a high-to-low phenotype; arrows indicate CD161_Low_ population, and boxes indicate CD161^+^ population (#*P*<0.05; ##*P*<0.01 vs SHRSP GD 18). CD161^+^ populations exhibited high expression of cytotoxic granzyme B and CD161_Low_ population exhibited significantly lower expression of granzyme B (**E**–**F**; **P<0.01 vs CD161_Low_). Data were analyzed by 1-way ANOVA followed by post hoc Tukey test.

Etanercept treatment in the SHRSP significantly reduced the number of CD161^+^ NK cells present in the SHRSP placenta (Figure 6A) but not in the maternal circulation (Figure 6B). Within the placental CD161^+^ cells in SHRSP animals only, we identified a CD161 low (CD161_Low_) population and a CD161 positive (CD161^+^) population (Figure 6C). Reduction of CD161^+^ cells in the placenta from SHRSP treated with etanercept was associated with a significant increase in the CD161_Low_ population (Figure 6D). The CD161^+^ population had a characteristically cytotoxic phenotype where the majority of these cells stained positively for intracellular granzyme B from placenta in both SHRSP and SHRSP treated with etanercept. In contrast, the CD161_Low_ population had a significantly decreased proportion of granzyme B expression from placenta in both SHRSP and SHRSP treated with etanercept (Figure 6E and 6F). Therefore, etanercept treatment reduces CD161^+^ cells in the SHRSP placenta by reducing the expression of CD161 receptor, which is associated with a reduction in granzyme B production.

## Discussion

Etanercept treatment has beneficial effects on the placenta and litter size of the SHRSP through the improvement of uterine artery function and uteroplacental blood flow. Etanercept antagonizes signaling from the excess TNF-α produced by a pregnancy-dependent increase in CD161^+^ NK cells found in the SHRSP and has direct effects on the placenta where it minimizes cytotoxic granzyme B from these CD161^+^ cells.

Etanercept has previously been shown to reduce blood pressure in a rat model of preeclampsia.^21,25^ In contrast, it had no effect on blood pressure in nonpregnant hypertensive female rats.^26^ These differing results show that etanercept has context-dependent effects on blood pressure, perhaps related to the extent or localization of inflammation. The average SBP reduction of 11.5 mm Hg in pregnant SHRSP treated with etanercept is comparable to the reduction in blood pressure seen in other rat models.^21,25^ As etanercept does not completely normalize blood pressure in the SHRSP; there must be other as-yet-unknown mechanisms that contribute to the hypertension we observe in this model. In this study, the decrease in SBP is not present until after GD 12 in the SHRSP. This coincides with the development of the mature uteroplacental unit by GD 12, suggesting that the placenta may be the source of excess TNF-α. Reducing maternal blood pressure during pregnancy using antihypertensive agents has been related to fetal growth restriction.^27^ Importantly, in this study, the significant reduction in SBP in late pregnancy has no negative impact on fetal growth as no significant difference in fetal weight was observed between vehicle- and etanercept-treated SHRSP. In fact, pregnancy outcome is improved in SHRSP treated with etanercept where there is a significant increase in litter size and a decrease in dams presenting with resorptions at GD 18. Etanercept treatment has been previously shown to reduce spontaneous pregnancy loss in a model of abnormal maternal inflammation during pregnancy.^28^

Etanercept treatment improves endothelium-dependent vasorelaxation in SHRSP uterine arteries. TNF-α has a well-defined role in endothelial dysfunction by diminishing the bioavailability of vasodilator nitric oxide through the downregulation of the expression of the eNOS (endothelial NO synthase) pathway^29,30^ and through increased superoxide production.^31^ Etanercept also significantly reduces the uterine artery vasocontractile response to noradrenaline in our model. Changes in the vascular response to noradrenaline in etanercept-treated SHRSP could indicate changes in sympathetic nervous system activity in these animals. As a crude measure of sympathetic drive, we analyzed heart rate variability that showed a significant reduction over pregnancy in etanercept-treated SHRSP (data not shown). However, these results are difficult to interpret taking into account the change in heart rate over pregnancy. TNF-α has been shown to induce the production of vasoconstrictor endothelin-1^32^ and can alter calcium handling.^33^ In the context of the experiment presented here, etanercept may have a role in the downregulation or desensitization of contractile adrenoreceptors to blunt the response to noradrenaline. In vascular function studies using resistance vessels from ovarectomized female rats where systemic TNF-α is elevated, etanercept treatment reduces contractility and improves endothelium-dependent vasorelaxation.^34^ In keeping with the improvement in uterine artery function in the SHRSP, uteroplacental blood flow is partially restored with etanercept treatment. Correction of deficient uteroplacental blood flow during pregnancy using a TNF-α antagonist has not previously been shown in the literature. However, plasma TNF-α has been shown to have a positive correlation with abnormal uterine artery resistance index and the presence of notching in humans.^35^ We have previously hypothesized that deficient uteroplacental perfusion in SHRSP leads to the premature utilization of glycogen stores in the placenta.^13^ In agreement, improvement of uteroplacental blood flow in etanercept-treated SHRSP restores the presence of placental glycogen cells at GD 18.

In experiments into the source of excess TNF-α in pregnant SHRSP, we found that the number of CD3^−^ CD161^+^ NK cells was increased in a pregnancy-dependent manner in the SHRSP but not in the control WKY. NK cells are part of the innate immune system with potent cytotoxic ability. In the context of hypertension, the NK cell gene locus has been shown to confer susceptibility to L-NAME (*N*_ω_-nitro-l-arginine methyl ester hydrochloride)–induced hypertension in mice^36^ and NK cells have been shown to play a role in endothelial dysfunction.^37^ The study of NK cells in pregnancy has principally focused on the uterine-specific population of these cells, which plays an important role in remodeling the uterine spiral arteries in response to pregnancy.^38^ Changes in peripheral NK cells do not directly mirror the status of uterine-specific NK cells; therefore, the 2 populations should be considered separately.^39^ In humans, the number of peripheral NK cells and their cytotoxicity are increased relative to nonpregnant levels in the first trimester followed by a decline in late pregnancy.^40^ Alterations in peripheral NK cells have mostly been associated with recurrent pregnancy loss and infertility; however, these findings are based on relatively small, observational studies.^39^ Other small studies have also explored a proinflammatory shift in NK cells in preeclampsia.^41^ We identified that CD161^+^ cells from SHRSP produced excess TNF-α relative to control WKY. Although we cannot conclusively prove that excess TNF-α is not being produced by other cell types; intracellular staining for TNF-α was not significantly different in CD3^+^ or other CD45^+^ cells. TNF-α is also produced by invasive extravillous trophoblasts; however, at GD 18 of pregnancy, trophoblasts do not exhibit potent invasive behavior.^42^

The presence of total CD161^+^ cells was increased in placenta from SHRSP and SHRSP treated with etanercept, whereas this was minimal in the WKY placenta. Therefore, a currently unknown TNF-α–independent mechanism exists, which recruits NK cells to the placenta of SHRSP. Within this placental CD161^+^ cell population, we have shown that etanercept induces a significant reduction in CD161 expression, which is associated with a reduction in granzyme B production. Conversely, this infers that TNF-α signaling is a critical step involved in CD161 upregulation and granzyme B production in NK cells. Previously, TNF-α has been shown to work in concert with interferon-γ to promote the cytotoxic activity of NK cells.^43^ The CD161 antibody used in this study detects the NKRP1A and NKRP1B cell surface receptors selective for NK cells. The killer ability of NK cells is protected by a sophisticated mechanism of activating and inhibitory receptors, whereby the resulting action is determined by the balance of these signals. NKRP1A^44^ is an activating receptor and NKPR1B an inhibitory receptor in the rat.^45^ CD161 is conserved in mice where it is also selective for NK cells^46^ and in humans where it is only expressed on a subset of NK cells.^47^ Although we have shown that CD161^+^ NK cells are associated with a cytotoxic phenotype, future studies should examine the balance of receptors on the NK cell surface in hypertensive pregnancy to further define their role in pathology.

Pregnancies complicated by chronic hypertension are associated with an increased risk of developing serious pregnancy-related complications affecting both the mother and fetus.^3^ Treating women who are pregnant is challenging because of the potential effects on the fetus, and no cure exists for more severe hypertensive complications of pregnancy. Small amounts of etanercept have been shown to cross the placental barrier in humans; a ratio between 14:1 and 30:1 (maternal serum:cord blood) has been reported in the literature.^48,49^ Etanercept has been used during pregnancy in women with inflammatory bowel disease and rheumatoid arthritis, where small studies have reported no adverse pregnancy outcomes,^50^ and in pilot studies as a treatment in early pregnancy for women with recurrent spontaneous miscarriage where it has a promising therapeutic effect.^51,52^ More convincing evidence of the safety of anti–TNF-α therapy in pregnancy will come from the ongoing Organisation of Teratology Information Specialists Autoimmune Diseases in Pregnancy Project (NCT01086059) expected to be reported in early 2017.^53^

## Perspectives

Excess TNF-α associated with an increase in CD161^+^ NK cells plays a causative role in abnormal vascular adaptation and placental biology in this preclinical model of chronic hypertension in pregnancy. Etanercept ameliorates the adverse pregnancy outcomes associated with this model, including direct effects on the placenta where it reduces CD161 expression and granzyme B production from NK cells. Thus, etanercept should be considered in future studies as a promising therapeutic avenue for pregnancies complicated by hypertension.

## Sources of Funding

The study was funded by grants from the European Union (EU-MASCARA; project reference 278249) and the Scottish Government’s Chief Scientist Office (reference ETM/196). H.Y. Small is funded by a British Heart Foundation Student Fellowship (FS/12/66/30003).

## Disclosures

None.

## Supplementary Material

## Novelty and Significance

### What Is New?

- This is the first study to examine the effect of etanercept on the uterine vasculature during pregnancy.
- We demonstrated that a tumor necrosis factor-α–targeted strategy reduces blood pressure and increases litter size in the stroke-prone spontaneously hypertensive rat relative to the control Wistar–Kyoto rat.
- CD161^+^ natural killer cells which produce excess tumor necrosis factor-α accumulate in the placenta of the stroke-prone spontaneously hypertensive rat relative to the Wistar–Kyoto rat. Etanercept reduces the expression of cytotoxic granzyme B in natural killer cells found within the placenta of the stroke-prone spontaneously hypertensive rat.

### What Is Relevant?

- Chronic hypertension during pregnancy is an increasing problem and confers significant risk for both maternal and fetal morbidity and mortality.
- The role of inflammatory cytokines and immune cells has been well characterized in the remodeling of the small spiral arteries of the uterus in response to pregnancy. Here we suggest they may also play a role in remodeling larger arteries.
- Etanercept, which is currently not contraindicated for use during pregnancy, presents itself as a promising drug for hypertensive complications of pregnancy in selected cases.

### Summary

TNF-α associated with an increase in CD161^+^ NK cells plays a causative role in abnormal vascular adaptation and placental biology in this pre-clinical model of chronic hypertension in pregnancy. Treatment with etanercept ameliorates the adverse pregnancy outcomes associated with this model; including direct effects on the placenta where it reduces CD161 expression and granzyme B production from NK cells. Thus, etanercept should be considered in future studies as a promising therapeutic avenue for pregnancies complicated by hypertension.
