# Supplementary material for: Role of Tumor Necrosis Factor-α and Natural Killer Cells in Uterine Artery Function and Pregnancy Outcome in the Stroke-Prone Spontaneously Hypertensive Rat
Source: Hypertension. 2016 Oct 12;68(5):1298–307. doi: 10.1161/HYPERTENSIONAHA.116.07933 (PMC5058643; doi:10.1161/HYPERTENSIONAHA.116.07933)
Supplement: Supplementary file 1 [file hyp-68-1298-s001.docx]

**SUPPLEMENTAL MATERIAL: THE ROLE OF TNFα AND NATURAL KILLER CELLS IN UTERINE ARTERY FUNCTION AND PREGNANCY OUTCOME IN THE STROKE PRONE SPONTANEOUSLY HYPERTENSIVE RAT**

Heather Yvonne Small MRes BSc (Hons)^1^, Ryszard Nosalski MSc_1_^, 2^, Hannah Morgan MRes BSc (Hons)^1^, Elisabeth Beattie MSc BSc (Hons)^1^, Prof. Tomasz J Guzik MD PhD FRCP(Glasg)^1^, Dr. Delyth Graham PhD^1^, Prof. Christian Delles MD FAHA^1^

^1^ Institute of Cardiovascular and Medical Sciences, University of Glasgow, G12 8TA.

^2^ Department of Internal Medicine, Jagiellonian University Medical College, Cracow, Poland

**Corresponding Author:**

Ms. Heather Small

Address: BHF GCRC, 126 University Place, Glasgow, G128TA

Telephone Number: 01413305189

Email Address: h.small.1@research.gla.ac.uk

**Supplemental Methods**

**Radiotelemetry**

Systolic (SBP) and diastolic blood pressure (DBP), heart rate and activity were directly monitored using the Dataquest V telemetry system (Data Sciences International, Sheffield, UK) in WKY, SHRSP and SHRSP treated with etanercept (n=4 in each group). Prior to implantation, the radiotelemetry transmitter was checked for calibration to be accurate within ± 6 mmHg. Animals were allowed 2 weeks to recover before time mating.

**Doppler Ultrasound**

Doppler waveform recordings were used to assess uterine artery blood flow in WKY, SHRSP and SHRSP treated with etanercept (n=6 in each group). Rats were anaesthetized using an inhalational anaesthetic (1.5 % isoflurane/oxygen) throughout the procedure. Ultrasound gel was applied to exposed skin as an ultrasound coupling medium. Rats were imaged trans-abdominally using an Acuson Sequoia C256 imager fitted with a 15-MHz linear array transducer (Siemens, Surrey, UK). Peak systolic velocity (PSV) and end diastolic velocity (EDV) was measured from 6 consecutive cardiac cycles. Resistance index (RI) was calculated as (RI = [PSV- EDV]/PSV).

**Wire Myography**

Rings from the main uterine artery from pregnant (GD18) WKY, SHRSP and SHRSP treated with etanercept (n=6 in each group) were prepared consistently from arterial segments located closer to the vagina than the ovary from the most pregnant horn then harvested in calcium free physiological salt solution (PSS) (0.25 M NaCl, 0.001 M KCl, 2 mM MgSO4, 50 mM NaHCO3, 2 mM KH2PO4, 1 mM glucose, 0.5 ml of 23 mM EDTA). Uterine artery rings (1.8 -2.0 mm in length) were mounted on two stainless steel wires on a four channel small vessel myograph (AD Instruments, Oxford, UK) in PSS (0.25 M NaCl, 0.001 M KCl, 2 mM MgSO4, 50 mM NaHCO3, 2 mM KH2PO4, 1 mM glucose; 2.5 mM CaCl2). Vessels were normalized and subject to a wake-up procedure as described previously (5). To establish the vessel’s contractile response, noradrenaline (Sigma-Aldrich, Dorset, UK) was added at the following increasing concentrations: 1x10-9, 1x10-8, 1x10-7, 1x10-6, 1x10-5 and 3x10-5 M. To determine the vessel’s endothelium dependent relaxation response, vessels were pre-constricted with 1x10-5 M noradrenaline followed by the addition of carbachol (Sigma-Aldrich, Dorset, UK) at the following increasing concentrations: 1x10-8, 1x10-7, 1x10-6 and 1x10-5 M.

**Pressure Myography**

Uterine artery rings from pregnant (GD18) WKY, SHRSP and SHRSP treated with etanercept (n=6 in each group) were mounted for pressure myography in calcium free PSS. The pressure myograph system (Danish Myo Technology, Aarhus N, Denmark) was set up and equilibrated according to manufacturer’s instructions. Arteries were tied to two glass cannula using nylon thread. Any side branches of the artery were also tied closed with nylon thread. Vessels were maintained at 37 ºC and 95 % O2 and 5 % CO2 throughout the experiment. After equilibration at 70 mmHg for one hour, the vessels were subject to increasing intraluminal pressure: 10, 20, 40, 60, 80, 100 and 110 mmHg. Measurements of internal and external diameter were taken after five minutes at each pressure. Wall thickness was calculated as [(external diameter - internal diameter) / 2]. Cross sectional area was calculated as [(π/4) x (external diameter² - internal diameter²)].

**Identification of Implantation Sites**

Rats were anaesthetized (2.5% isoflurane) throughout the procedure. 250 μl of 1% Evans’ blue dye (Sigma-Aldrich, Dorset, UK) in PBS was injected into the exposed femoral vein. After 10 minutes, the animal was sacrificed under terminal anaesthesia and the uterine horn was excised. Implantation sites were determined by clear bands of positive blue staining.

**Placental Tissue Explants**

Placental tissue was harvested in ice cold PBS from WKY, SHRSP and SHRSP treated with etanercept (n=5 in each group). Under sterile conditions, the tissue was dissected into pieces <5mm in ice cold DMEM F-12 Media +0.5% FBS +1% penicillin/streptomycin (Thermo Fisher Scientific, Paisley, UK). The tissue pieces were split evenly between 2 wells of a 24 well plate (Thermo Fisher Scientific, Paisley, UK) in 1ml of media. After 1 hour, the explants were washed in PBS and replaced with a fresh 1ml of media. Explants were incubated for 20 hours at 37 °C after which the media was collected and stored at -80 °C.

**ELISA**

TNFα in plasma, urine and explant media from WKY, SHRSP and SHRSP treated with etanercept (n=6 in each group for plasma and urine measurements; n=5 in each group for placental explant media measurements) was measured using a commercially available kit (R&D Systems, Abingdon, UK) according to manufacturer’s instructions.

**Leukocyte Isolation**

Leukocytes were isolated on the day of collection. Tissue from 3 uteroplacental units from pregnant (GD18) WKY, SHRSP and SHRSP (n=6 in each group) treated with etanercept were dissected, perfused with PBS (Thermo Fisher Scientific, Paisley, UK) then harvested in ice-cold PBS (Thermo Fisher Scientific, Paisley, UK). The placental tissue was disrupted using scissors to produce a single cell suspension which was passed through a 70 μm cell strainer (BD Bioscience, Oxford, UK). Leukocytes were isolated from blood collected in heparinized tubes (BD Bioscience, Oxford, UK) from non-pregnant (n=4 in each group) and pregnant (GD18) (n=5-6 in each group) WKY and SHRSP by density gradient centrifugation with Histopaque® (Sigma-Aldrich, Dorset, UK). Cells were washed once (1300 rpm for 6 minutes) and resuspended in 3ml of 2% FBS/PBS (FACS buffer) and placed on ice. Cells were counted using a haemocytometer and 1 x 10^6^ leukocytes were subject to staining procedure. Cells were used for staining on the day of tissue collection.

**Extracellular Flow Cytometry Panel**

Isolated leukocytes (details in supplemental material) from WKY (NP: n=4, GD18: n=5), SHRSP (NP: n=4, GD18: n=5) and SHRSP treated with etanercept (n=6) were stained using Zombie® live/dead dye (Biolegend, London, UK) for 15 minutes at room temperature in the dark followed by staining for extracellular markers using flurochrome conjugated primary antibodies for 20 minutes on ice in the dark. Two panels of antibodies were used. Panel 1: anti-CD45-PerCP/Cy5.5, anti-CD3-APC, anti-CD4-PE/Cy7, anti-CD8-FITC and anti-CD161-PE, (Biolegend, London, UK). Panel 2: anti-CD45-PerCP/Cy5.5, anti-CD103-AlexaFluor®647, anti-CD4-PE/Cy7, anti-RT1B-FITC (Biolegend, London, UK) and anti-HIS36-PE (BD Bioscience, Oxford, UK). Cells were washed and resuspended in FACS buffer for immediate analysis using a BD FACSCanto II machine (BD Bioscience, Oxford, UK) with BD FACSDIVA™ software

**Intracellular Staining**

Cells from pregnant (GD18) WKY, SHRSP and SHRSP treated with etanercept (n=4 in each group) were cultured in RPMI 1640 media +10% FBS +1% penicillin/streptomycin (Thermo Fisher Scientific, Paisley, UK) then stimulated using a cell stimulation cocktail (BD Bioscience, Oxford, UK) at 2 µl/ml media and incubated at 37 °C for 4 hours. Cells were harvested after this time and stained using Zombie® live/dead dye (Biolegend, London, UK) for 15 minutes at room temperature in the dark followed by staining for extracellular markers using flurochrome conjugated primary antibodies for 20 minutes on ice in the dark (anti-CD45-PerCP/Cy5.5, anti-CD3-APC and anti-CD161-PE; Biolegend, London, UK). Cells were then fixed in intracellular fixation buffer (eBioscience, Hatfield, UK) for 45 minutes and washed in 1% permeabilisation buffer (eBioscience, Hatfield, UK) at 1100rpm for 6 minutes at 4 °C. Cells were then stained according to manufacturer’s instructions for intracellular TNFα (anti-TNFα-PE-Cy7; eBioscience, Hatfield, UK) and Granzyme-B (anti-granzyme-B-FITC antibody, Biolegend, London, UK) at room temperature for 30 minutes in the dark. Cells were washed and resuspended in FACS buffer for immediate analysis using a BD FACSCanto II machine (BD Bioscience, Oxford, UK) with BD FACSDIVA™ software where 10,000 CD45+ events was used as a cut-off in each sample.

**Histology**

Uteroplacental tissue from WKY, SHRSP and SHRSP treated with etanercept (n=6 in each group) was fixed for 24 hours in 10% formalin. Paraffin sections of 5 µm were used for staining. Immediately prior to staining, slides were deparaffinised and rehydrated through an ethanol gradient into distilled H2O. Periodic acid – Schiff (PAS) stain was used to quantify glycogen cell content in the placenta. Slides were incubated in 1% periodic acid/dH2O (Sigma-Aldrich, Dorset, UK) for 10 minutes followed by 1 minute under running tap water. Slides were then stained with Schiff’s reagent (Sigma-Aldrich, Dorset, UK) for 10 minutes. This was followed by 1 minute under running tap water and dehydration through an ethanol gradient and mounted using DPX (Sigma-Aldrich, Dorset, UK). Sections were viewed using light microscopy where 8-10 images were taken at 4x objective and laced together using Microsoft Image Composite Editor 2.0 Positive staining for histology was determined using a threshold quantification method in Image J (National Institutes of Health, Bethesda, USA). Sections were analysed by an operator who was blinded to the identity of the slides. The % positive staining was determined as (the amount of pixels over the given threshold ratio/the total number of pixels) x 100.

**Gene Expression**

Uteroplacental tissue was snap frozen in liquid nitrogen and stored at -80 °C until use. RNA was extracted using the miRNeasy kit (Thermo Fisher Scientific, Paisley, UK) according to manufacturer’s instructions. RT-PCR was used to prepare complimentary DNA (cDNA). RT-PCR was performed using the Taqman® Reverse Transcription Kit (Thermo Fisher Scientific, Paisley, UK) according to manufacturer’s instructions with 1 µg RNA input. The reaction was run on a Multi Block System Satellite 0.2 Thermo Cooler (Thermo Fisher Scientific, Paisley, UK) on the following settings: 25 °C 10 min, 48 °C 30 min, 95 °C 5 min. qPCR was used to quantify gene expression. Reactions were set up using the following reagents: Taqman® Universal Mastermix (Thermo Fisher Scientific, Paisley, UK), nuclease free H_2_O (Thermo Fisher Scientific, Paisley, UK) and relevant Taqman® probe (Thermo Fisher Scientific, Paisley, UK). The reaction was run on a QuantiStudio® 12K Flex (Thermo Fisher Scientific, Paisley, UK) at the following settings: 95 °C, 15 min; followed by 40 cycles of 95 °C, 15 s; 60 °C, 1 min. Ct values were analysed using the 2^(-ΔΔCt)^ method, with ΔCt indicating normalisation to the housekeeper β-actin (*Actb*). This experiment was run using the Taqman® system (Thermo Fisher, Paisley, UK) where Tnfr1 expression was assessed using the probe Rn01492348_m1 (Thermo Fisher, Paisley, UK) which was normalised to the housekeeper; Gapdh Rn01462661_g1 (Thermo Fisher, Paisley, UK).

**Supplemental Figures & Figure Legends**

**Supplementary Figure S1: Etanercept does not significantly alter TNFα levels in maternal plasma or from placental explants at GD18.** TNFα was measured in plasma (A) (n=6) and media from placental explants (B) (n=5) using ELISA at GD18 in WKY, SHRSP and SHRSP treated with etanercept. SHRSP and SHRSP treated with etanercept showed elevated levels of TNFα in both maternal plasma and in media from placental explants (* p>0.05 vs. GD18 WKY).

**Supplementary Figure S2: Heart rate and activity before and during pregnancy in the WKY, SHRSP and SHRSP treated with Etanercept.** Heart rate (A) was not significantly altered between the groups. Activity (B) was significantly decreased in SHRSP and SHRSP treated with Etanercept relative to WKY (* p<0.05 vs. WKY analysed by two-way ANOVA).

**Supplementary Figure S3: Blastocyst implantation is not altered between WKY and SHRSP.** Blastocyst implantation was determined at GD6 in untreated SHRSP and WKY (n=3-4) using Evans’ blue dye infusion (A) under terminal anaesthesia. There was no significant difference between WKY and SHRSP in the number of implantation sites counted (B).

**Supplementary Figure S4: Maternal, fetal and placental weight are unaffected by etanercept treatment in the SHRSP.** Fetal (A) and placental (B) weight did not show any significant differences between strains at GD18 (n= 6-12). Maternal weight gain (B) during pregnancy was significantly decreased in both SHRSP and SHRSP treated with etanercept relative to WKY (data analysed by area under the curve followed by one way ANOVA; ** p<0.01 vs. WKY).

**Supplementary Figure S5: Etanercept treatment does not alter uterine artery size in the SHRSP.** Isolated uterine artery properties were assessed using pressure myography to measure external (A) and internal (B) diameter, cross-sectional area (C) and wall thickness (D) in etanercept treated SHRSP, vehicle treated SHRSP and untreated WKY (n=6) at GD18 of pregnancy. External and internal diameter as well as cross-sectional area was significantly decreased in both untreated and etanercept treated SHRSP (data analysed by area under the curve followed by one way ANOVA; * p<0.05 vs. WKY; ** p<0.01 vs. WKY).

**Supplementary Figure S6: Etanercept treatment does not alter mesenteric artery function in the SHRSP.** Isolated mesenteric artery function was assessed using wire myography in WKY (n=4), SHRSP and SHRSP treated with etanercept (n=6) at GD18 of pregnancy. There was a trend for etanercept to shift the contractile response to noradrenaline (A). Etanercept treatment did not significantly improve endothelium dependent vasorelaxation to carbachol (* p<0.05 vs. GD18 WKY) (B). Data analysed by calculating area under the curve followed by one way ANOVA and post-hoc Tukey test.

**Supplementary Figure S7: Full flow cytometry panel in maternal blood from pregnant WKY, SHRSP and SHRSP treated with etanercept.** A flow cytometry panel was used to quantify immune cell sub-types in the maternal blood from pregnant (GD18) WKY, SHRSP and SHRSP treated with etanercept (n=5-6). The percentage of T cells (A) in the maternal blood was marginally but significantly increased in SHRSP and SHRSP treated with etanercept relative to the WKY, however there was no significant difference between the balance of helper (B) and cytotoxic T cells (C). Both NK cells (D) and NK-T cells (E) were significantly increased in SHRSP and SHRSP treated with etanercept relative to the WKY. There were no significant alterations in the presence of dendritic cells. Data was analysed using one way ANOVA followed by a post-hoc Tukey’s test where * p<0.05, ** p<0.01, *** p<0.0005 vs. WKY GD18.

**Supplementary Figure S8: Full flow cytometry panel in placenta from pregnant WKY, SHRSP and SHRSP treated with etanercept.** A flow cytometry panel was used to quantify immune cell sub-types in placenta from pregnant (GD18) WKY, SHRSP and SHRSP treated with etanercept (n=5-6). T cells (A) and Th cells (B) were significantly increased in SHRSP treated with etanercept relative to WKY. NK cells (D) were significantly increased in the SHRSP relative to the WKY and SHRSP treated with etanercept. NK-T cells (E) were significantly increased in SHRSP and SHRSP treated with etanercept. The detection of dendritic cells (F) was variable and not measurable in all samples. There was a trend for etanercept to reduce the number of macrophages in the SHRSP (G). Data was analysed using one way ANOVA followed by a post-hoc Tukey’s test where * p<0.05, **p<0.01 vs. WKY GD18 and ## p<0.01 vs. SHRSP.

**Supplementary Figure S9: Gating strategy for extracellular immune cell panel.**

**Supplementary Figure S10: TNFα production from CD3+ cells and other CD45+ cells is not significantly different between WKY and SHRSP.** Intracellular staining was used to quantify TNFα production from immune cells in the maternal blood and placenta from WKY and SHRSP. In placenta (A-B) and maternal blood (C-D) there was no significant difference in TNFα production from CD3+ or other CD45+ cells, only in CD161+ cells (* p<0.05, ** p<0.01 vs. WKY GD18). Data analysed using Student’s t test.
